# An HPLC-MS Characterization of the Changes in Sweet Orange Leaf Metabolite Profile following Infection by the Bacterial Pathogen *Candidatus* Liberibacter asiaticus 

**DOI:** 10.1371/journal.pone.0079485

**Published:** 2013-11-05

**Authors:** Faraj M. Hijaz, John A. Manthey, Svetlana Y. Folimonova, Craig L. Davis, Shelley E. Jones, José I. Reyes-De-Corcuera

**Affiliations:** 1 IFAS, Citrus Research and Education Center, University of Florida, Lake Alfred, Florida, United States of America; 2 U.S. Horticultural Research Laboratory, USDA, ARS, Fort Pierce, Florida, United States of America; 3 Department of Plant Pathology, University of Florida, Gainesville, Florida, United States of America; Institute for Plant Protection (IPP), CNR, Italy

## Abstract

Huanglongbing (HLB) presumably caused by *Candidatus* Liberibacter asiaticus (*C*Las) threatens the commercial U.S. citrus crop of an annual value of $3 billion. The earliest shift in metabolite profiles of leaves from greenhouse-grown sweet orange trees infected with *C*las, and of healthy leaves, was characterized by HPLC-MS concurrently with PCR testing for the presence of *C*las bacteria and observation of disease symptoms. Twenty, 8-month-old ‘Valencia’ and ‘Hamlin’ trees were grafted with budwood from PCR-positive HLB source trees. Five graft-inoculated trees of each variety and three control trees were sampled biweekly and analyzed by HPLC-MS and PCR. Thirteen weeks after inoculation, *C*las was detected in newly growing flushes in 33% and 55% of the inoculated ‘Hamlin’ and ‘Valencia’ trees, respectively. Inoculated trees remained asymptomatic in the first 20 weeks, but developed symptoms 30 weeks after grafting. No significant differences in the leaf metabolite profiles were detected in *C*las-infected trees 23 weeks after inoculation. However, 27 weeks after inoculation, differences in metabolite profiles between control leaves and those of *C*las-infected trees were evident. Affected compounds were identified with authentic standards or structurally classified by their UV and mass spectra. Included among these compounds are flavonoid glycosides, polymethoxylated flavones, and hydroxycinnamates. Four structurally related hydroxycinnamate compounds increased more than 10-fold in leaves from ‘Hamlin’ and ‘Valencia’ sweet orange trees in response to *C*las infection. Possible roles of these hydroxycinnamates as plant defense compounds against the *C*las infection are discussed.

## Introduction

Citrus greening disease, also known as huanglongbing (HLB), is the most destructive citrus disease worldwide [1]. It is believed to be caused by the bacterium *Candidatus* Liberibacter asiaticus (Clas), which is transmitted by the Asian citrus psyllid (*Diaphorina citri*, ACP) [1]. Recently, HLB was discovered in the largest citrus growing regions in the USA and Brazil [2]. HLB symptoms are displayed on different parts of the plant; the leaves are characterized by a yellow blotchy mottle, or asymmetrical chlorosis, and the fruits are under-developed, lopsided, and green colored with aborted or stained seeds [3]. As the disease severity increases, both yield and fruit quality decrease. Yield reduction due to fruit drop can reach 30 to 100% [1]. 

*C*las bacterium is not the only type of *Candidatus* Liberibacter that causes a destructive disease in commercial crops. In fact, the phloem-limited bacterium (*Candidatus* Liberibacter solanacearum), which is transmitted by the potato psyllids *Bactericera cockerelli* Sulc. (Hemiptera: Triozidae), can negatively affect a range of vegetable hosts [4]. In potatoes, *Candidatus* Liberibacter solanacearum is associated with zebra chips (ZC) disease. The ZC symptoms include purple or yellow discoloration, leaf burn, aerial tubers, and early death [5].Tubers from infected plants showed brown discoloration after slicing and very dark blotches, stripes, or streaks upon frying [5]. 

The incubation period for HLB within citrus trees ranges from a few months to one or more years [1]. At about 3 months after inoculation, *C*las was detected in 70% of inoculated sweet orange and grapefruit seedlings [6], and severe asymmetrical yellowing of leaves was clearly observed 5–6 months after grafting. In a similar study [7], *C*las bacterium was detected in 60% of the ‘Valencia’ trees 1 month after inoculation, and typical HLB symptoms (chlorosis of leaves) were observed 6–8 months after inoculation. Quantification of the bacterium using qPCR showed that the *C*las bacterium was present in different parts of the infected plant; however, it was unevenly distributed [8]. Although the previous studies provided useful information about the etiology of the HLB disease, transmission, symptom development, and *C*las detection, they did not provide any information about the effect of *C*las on the health and the biochemical change in fruits and leaves of infected plants.

Studies of the effects of HLB on the orange juice encompassed investigations into the effects of HLB disease on the taste and phytochemical compositions of the fruits. An increase in the concentrations of limonoids, a number of alkaloids, hydroxycinnamates (HCAs), phenylalanine, and citrate have been observed in ‘Valencia’ orange juice samples obtained from *C*las-infected trees [9,10].

Early studies of the effects of HLB on the phytochemical profiles of citrus leaves targeted starch [11] and gentisic acid [12]. More recent studies have characterized part of the metabolome of leaves from *C*las-infected and healthy citrus trees using high performance liquid chromatography-mass spectrometry (HPLC-MS) [13,14], capillary electrophoresis with photodiode array detection [15], and gas chromatography-mass spectrometry (GC-MS) [16]. However, these studies involved investigations of leaves obtained from commercial groves for which it was not possible to determine the time of initial infection, and for which multiple inoculations by psyllids likely occurred. Moreover, all of these studies focused on the metabolic difference between highly symptomatic and healthy leaves; hence, little has been achieved in characterizing potential early-occurring phytochemical markers in newly-infected pre-symptomatic leaves. The objective of our study was to evaluate biochemical changes that occur in the leaves of *C*las-infected sweet orange trees over time.

## Materials and Methods

### Tree inoculation with *Candidatus* Liberibacter asiaticus (*C*las) and sampling procedures

‘Valencia’ and ‘Hamlin’ (*Citrus sinensis* (L.) Osbeck) plants used in this study were grafted on Volkamer lemon (*Citrus limonia* Osbeck ‘Volkameriana’). Twenty plants (8–10 months old, 0.5‑1 m tall) from each variety were scion grafted with four pieces of budwood from PCR-positive HLB source trees. The grafts were 10 cm apart and the lowest graft was at about 15 cm above ground. Inoculated plants were kept in a USDA-APHIS approved secure greenhouse with the temperature controlled at 28–32°C. Plants were regularly watered twice a week and fertilized once a week using 20-10-20 fertilizer (Allentown, PA). For each variety, eight plants were grafted with disease-free budwood and were used as controls. Five inoculated trees of each variety and three control trees were sampled at 3, 23, 27, 29, 35, and 38 weeks after inoculation. The sampling plan was based on our previous observations in early studies [6,17]. From each tree, two leaves were randomly (regardless to symptoms) harvested from the same shoot, bagged, immediately placed on ice for transportation, frozen, and stored at -80°C.

### PCR analyses and leaf symptoms

The second leaf from each sampled tree was used for PCR analysis to check for the presence of the *C*las bacterium as described by Tatineni et al. [8]. Briefly, 250 mg of midrib leaf tissue were extracted in 2.5 ml extraction buffer (100 mM Tris-HCL pH 8.0; 50 mM EDTA; 500 mM NaCl; 10 mM dithiothreitol). A volume of 1500 µl was transferred to a 2.5 ml Eppendorf tube, and 100 µl 20% (w/v) SDS was added, and incubated at 65°C for 30 minutes. A 500 µl aliquot of 5 M potassium acetate (pH = 5.2) was added, mixed thoroughly, and incubated on ice for 20 minutes. DNA was recovered by centrifugation and precipitation with isopropanol and kept at -20°C overnight. Resuspended DNA was analyzed by conventional PCR using 0.2 μg of DNA template, 0.2 μM each primer (HLB-65 and HLB-66) [8], 0.25 mM dNTPs, 1× buffer (FBII; Takara Bio), and 0.125 μl (5 U/μl) of SpeedSTAR HS DNA polymerase. The amplification conditions were as follows: 94°C for 2 minutes; followed by 35 cycles at 94°C for 20 seconds, 54°C for 20 seconds, and 72°C for 60 seconds; and final extension at 72°C for 5 minutes. PCR reactions were analyzed in 1.0% agarose gel. DNA bands were visualized by ethidium bromide staining. All reactions were done in triplicate with positive, healthy, and water controls. Control and *C*las-inoculated trees were photographed before sampling, and sampled leaves were also photographed before storage to document any leaf symptoms (chlorosis, blotchy-mottled, or leaf yellowing).

### Sample preparation and HPLC-MS analyses of leaf metabolites

Methanol, dimethylsulfoxide, and formic acid were purchased from Sigma (St. Louis, MO). Diosmin, 6,8-di-C-glucosyl apigenin, 2′′-xylosylvitexin, 8-*C*-glucosyldiosmetin, luteolin-7-*O*-rutinoside, hesperidin, isosakuranetin rutinoside, sinensetin, nobelitin, 3,5,6,7,8,3′,4′-heptamethoxyflavone, tangeretin, feruloylputrescine, and 5-hydroxy-6,7,4′-trimethoxyflavone were obtained as authentic standards from the USDA (Fort Pierce, FL). 

One leaf from each sampled tree was removed from storage and a weighed portion (approximately 0.5 g) was ground with a mortar and pestle to a fine powder under liquid nitrogen. A 10-ml aliquot of methanol and dimethylsulfoxide (1:1) was added, and the sample was shaken overnight at 200 rpm in a cold chamber at 0°C using Innova 2100 platform shaker from New Brunswick Science (Edison, NJ). The samples were equilibrated to room temperature and filtered using 0.45 μm Titan 2 HPLC filters from Thermo Fisher Scientific (Pittsburg, PA). A 0.9-ml aliquot of the filtrate was spiked with 0.1 ml of 2500 ppm of 5-hydroxy-6,7,4′-trimethoxyflavone as an internal standard.

Metabolite profiles were analyzed by HPLC-MS, using Varian ProStar 210 pumps and a ProStar 410 autosampler controlled by Star software (ver. 6.41). Compound separations were achieved with a Waters Xbridge C8 column (4.6 × 150 mm i.d.). The mobile phase composition and the flow rate are given in Table 1. MS peak detection was achieved with a Leco APCI (atmospheric pressure chemical ionization) source coupled with a LECO Unique HT MS analyzer (St. Joseph, MI). MS parameters were as follows: positive atmospheric pressure ionization (+ APCI), interface temperature 99°C, nebulizer pressure 300 kPa, desolvation temperature 350°C, desolvation gas flow 2.0 L min^-1^, nozzle voltage 100 V, skimmer voltage 52 V, and quad RF voltage 165 V. Data processing was done with Leco ChromaTOF (ver. 4.0). Peak identifications were achieved by comparing retention times and mass spectra of sample peaks with those of authentic standards.

**Table 1:** Mobile phase composition and flow rate.

Time (min)	% A (0.5% formic acid, H_2_O)	% B (acetonitrile)	Flow (ml·min^-1^)
0	86	14	0.30
16	72	28	0.30
21	62	38	0.30
28	50	50	0.30
43	45	65	0.50
48	30	70	0.75
53	30	70	0.75
58	86	14	0.75
63	86	14	0.75
64	86	14	0.30
70	86	14	0.30

### Data processing and statistical analysis

Data was manually aligned using retention time and mass values. Data from infected and non-infected trees were normalized by dividing the area of each peak by the area of the internal standard (5-hydroxy-6,7,4′-trimethoxyflavone). Normalized data was analyzed using principal component analysis (PCA) using Unscrambler® X (www.camo.com). The mean of the normalized values (relative amount) of each compound in the *C*las-inoculated trees was also compared to the mean of the control trees at each sampling date using two tailed *t*-test with unequal replication using Microsoft Excel, and significant differences were reported at 95% confidence level. Data from *C*las-inoculated trees that were PCR-negative for the *C*las bacterium throughout the entire duration of the experiment were not included (one ‘Valencia’ and two ‘Hamlin’) in the statistical analyses, because those trees did not acquire the disease. In addition, analysis of covariance (ANCOVA) was carried on the whole data set for each cultivar, using time as a covariate, to study the effect of *C*las infection on the metabolites of sweet orange over time.

## Results and Discussion

### PCR analyses and leaf symptoms

Our preliminary PCR analysis showed that *C*las bacterium was detected in 3/9 and 5/9 of the inoculated ‘Hamlin’ and ‘Valencia’ plant, respectively, 13 weeks after inoculation. At 33 weeks after inoculation, 5/5 of the tested ‘Valencia’ and 4/5 of the tested ‘Hamlin’ plants were PCR positive (Table 2). Leaves from certain inoculated trees were PCR positive 13 weeks after inoculation, while leaves from the same trees but at different locations were PCR negative 21 and 29 weeks after inoculation, and then these trees were PCR positive 45 weeks after inoculation. These observations in both sweet orange varieties indicated that the bacterium was not evenly distributed in the inoculated trees. In addition, while some of the inoculated trees were PCR positive 13 weeks after inoculation, others did not show any PCR positive results before 45 weeks.

**Table 2:** PCR results for inoculated ‘Hamlin’ and ‘Valencia’ trees.

Weeks after inoculation	‘Hamlin’	‘Valencia’
3	0/5 ^a^	0/5
23	2/7	2/5
25	3/5	3/5
27	2/5	1/5
29	0/5	1/5
31	4/5	3/5
33	4/5	5/5
35	4/5	3/4
38	4/5	3/5

^*a*^Number of PCR positive plants over number of sampled plants at each sampling week.

**Figure 1:**
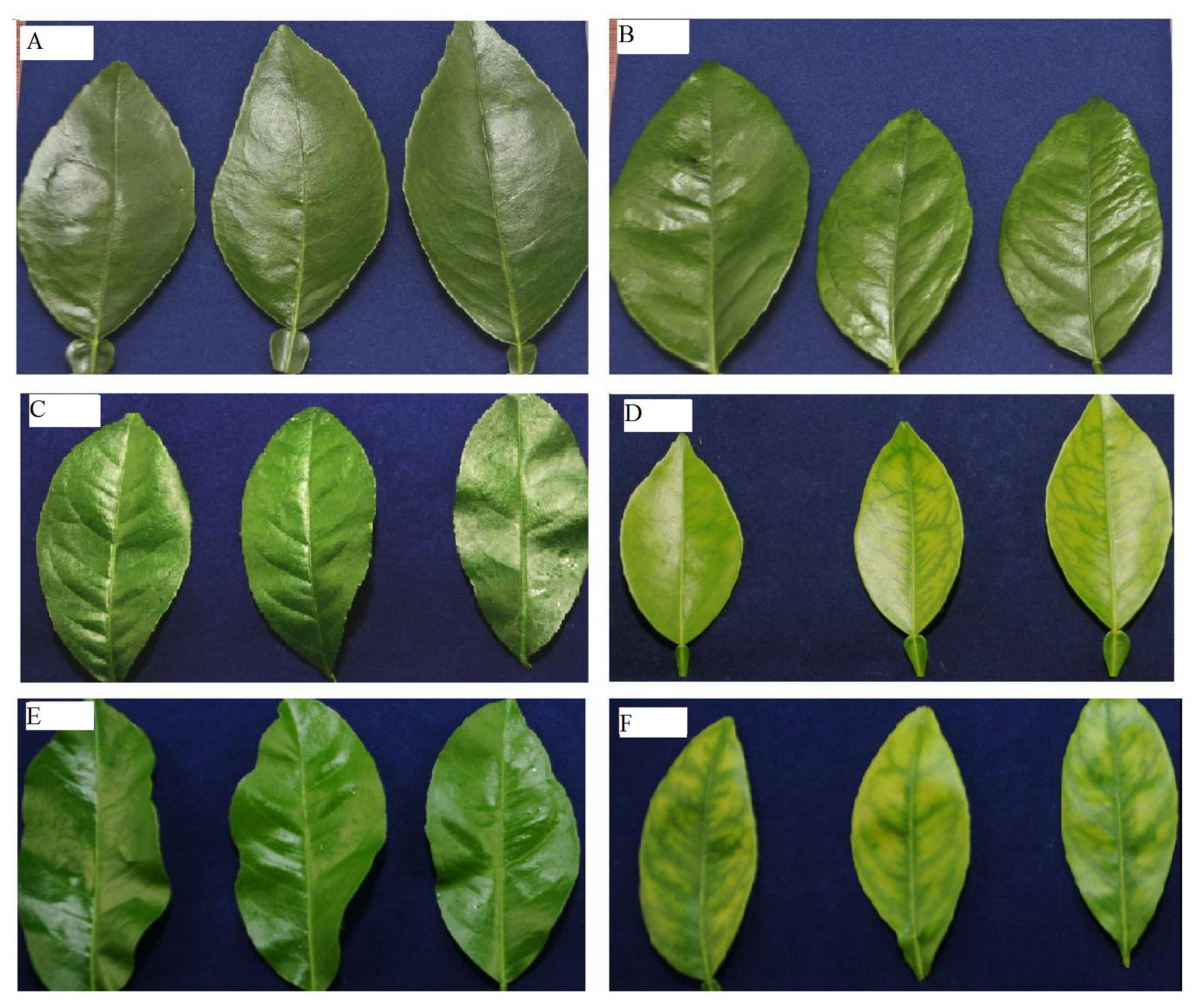
Progression of huanglongbing (HLB)-related symptoms in ‘*Candidatus* Liberibacter asiaticus’-inoculated ‘Valencia’ sweet orange seedlings. **A**) leaves from control plants 19 weeks after inoculation; **B**) leaves from HLB-grafted plants 19 weeks after inoculation; **C**) leaves from control plants 29 weeks after inoculation; **D**) leaves from HLB-grafted plants 29 weeks after inoculation; **E**) leaves from control plants 35 weeks after inoculation; **F**) leaves from HLB-grafted trees 35 weeks after inoculation.

**Figure 2:**
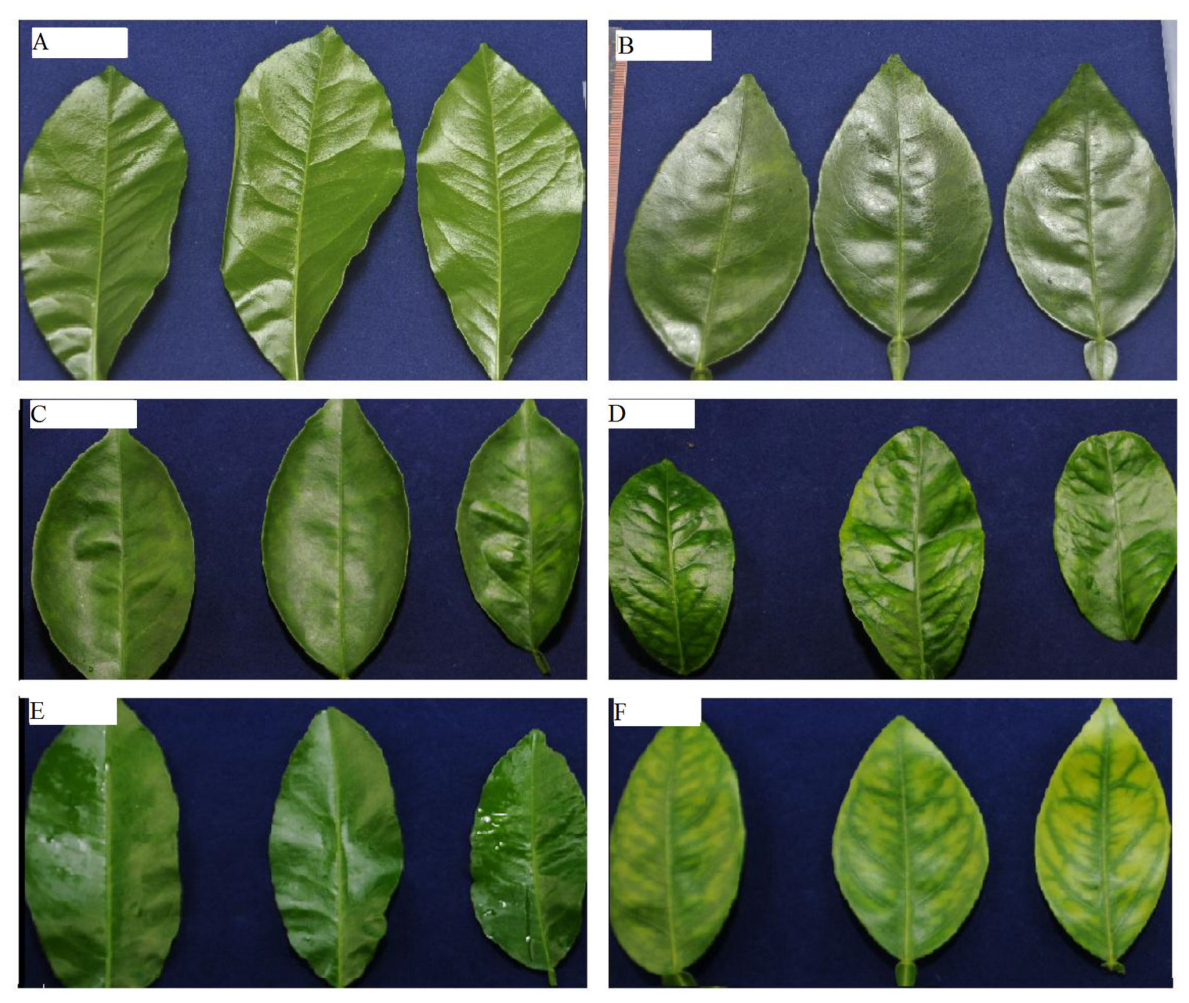
Progression of huanglongbing (HLB)-related symptoms in ‘*Candidatus* Liberibacter asiaticus’-inoculated ‘Hamlin’ sweet orange seedlings. **A**) leaves from control plants 19 weeks after inoculation; **B**) leaves from *C*Las-grafted plants 19 weeks after inoculation; **C**) leaves from control plants 29 weeks after inoculation; **D**) leaves from *C*Las-grafted plants 29 weeks after inoculation; **E**) leaves from control plants 35 weeks after inoculation; **F**) leaves from *C*Las-grafted trees 35 weeks after inoculation.

Leaves of the graft-inoculated trees remained asymptomatic in the first 19 weeks (Figure 1A-B and Figure 2A-B). Leaf symptoms characteristic of HLB started to develop in shoots of grafted plants at 19 weeks and were pronounced at 29 weeks after inoculation (Figure 1C-F and Figure 2 C-F). As with the uneven detection of the *C*las bacterium throughout the plants (Table 2), HLB leaf symptoms were also unevenly distributed throughout different portions of individual plants; some newly developed leaves on certain trees were highly symptomatic, while others were asymptomatic. These observations are consistent with those of Batool et al. [3] where HLB-symptoms occurred on individual shoots or branches, while other portions of the tree remained asymptomatic.

In this study, observations of leaf symptoms (Figure 1 and Figure 2), together with the PCR analyses, indicate that the *C*las bacterium in inoculated citrus trees are detected before the development of leaf symptoms, and that the probability of detecting *C*las is higher in the symptomatic leaves (31−37 weeks after inoculation). These findings are in close agreement with those reported in previous studies [6,8]. No *C*las bacterium was detected by qPCR in inoculated sweet orange and grapefruit 2 months after grafting, while 70% of the inoculated trees were PCR positive after 3 months [6]. Although *C*las bacterium has been found in different parts of infected citrus plants, qPCR showed that it was unevenly distributed [8]. In agreement with our study, no disease-related symptoms were observed at 3 months after inoculation; however, by 5−6 months, slight yellowing was observed [6]. In another study, *C*las bacterium was detected in 60% of the ‘Valencia’ trees one month after inoculation, and typical HLB symptoms (chlorosis of leaves) were observed 6−8 months after inoculation [7]. The results of this study together with the results of the previous studies [6,7,8] suggest that the observed HLB related symptoms and the PCR results are reproducible. However, the level of *C*las titer in the inoculation source, greenhouse temperature, grafting time, number of grafts per tree, applied nutrition, watering schedule, and the type of rootstock may result in slight variation between different studies.

Although the lack of visual leaf symptoms, prior to the detection of the *C*las bacteria in our study, suggests that the presence of the bacteria in the leaves may be necessary for the development of the visual symptoms, this hypothesis remains inconclusive. It is still possible that the bacterium could block the phloem of lower stems, thus producing chlorosis in the upper leaves [8], which may explain in part the inconsistent correlation between symptoms and PCR results.

### Metabolite profile analysis

The effects of *C*las graft-inoculation on disease symptom development in the ‘Hamlin’ and ‘Valencia’ trees were further explored by analyzing the changes in the leaf metabolite profiles, comprised mainly of phenolic flavonoids, hydroxycinnamates, and alkaloids (Table 3). The identification of a number of these compounds was achieved by observing co-elutions and matching MS and UV spectra with authentic standards. In addition, the UV spectra of these compounds were further compared to earlier published spectra [18]. For a number of the remaining compounds, structural classifications were made by inferences from their UV and mass spectra. Previous studies have shown that flavone-C- and -*O*-glycosides, primarily those of diosmetin (5,7,3′-trihydroxy-4′-methoxyflavone), apigenin (5,7,4′-trimethoxyflavone), and luteolin (5,7,3′,4′-tetrahydroxyflavone) are particularly abundant in leaves of many common citrus cultivars, including sweet orange [14,19,20]. Flavanone-*O*-glycosides are also abundant in citrus leaves, and compounds **20**-**22** in Table 3 are indicative of such compounds. Latter flavonoids in sweet orange occur as rutinosides [19] and are identified by sets of ions ([M+H]^+^, [M+H-(rhamnose-H_2_O)]^+^, [M+H-(rutinose-H_2_O)]^+^) readily detected in the positive APC-mass spectra of these compounds. Based on the above criteria and overlaps with standards, compounds **16**, **19**, **20**, and **22** were identified as luteolin-7-*O*- rutinoside, diosmin, hesperidin, and isosakuranetin rutinoside, respectively. In contrast, flavone-C-glycosides typically exhibit protonated molecular ions [M+H]^+^, and ion fragments corresponding to [M+H-nH_2_O]^+^ where n = 1–4. Several of the metabolites in Table 3 exhibited such patterns, including **8**, **12**, and **17**, and were identified as 6, 8-di-C-glucosylapigenin, 2′′-*O*-xylosylvitexin, and 8-*C*-glucosyldiosmetin, respectively. Several of the remaining unknown compounds listed in Table 3 also exhibited similar fragment ion patterns; but, due to an absence of standards, they remained unidentified. Also prevalent in the leaf chromatograms were compounds that exhibited UV and mass spectra of polymethoxylated flavones (PMFs) [20], and a number of these are listed in Table 3. Several other remaining compounds in Table 3 also exhibited UV spectra similar to the PMFs but had molecular weights 14 atomic mass units lower than the identified PMFs. These compounds may represent several of the 5-desmethyl PMFs previously reported in sweet orange tissues [20]. 

**Table 3:** APC mass-spectra in positive ion detection mode “*m/z*” and absorbance maximum (nm) for detected compounds.

Peak no.	RT (min)	Compound	“*m/z*” (+)	λ_max_ (nm)
1	5.2	Unknown	313.8	
2	5.3	Unknown	268	
3	5.4	Unknown	151	
4	10.2	Feruloylputrescine^a^	265/177	292/317
5	10.5	HCA^b^	386/195/177	325/230
6	12.5	HCA^b^	386/195/177	325/231
7	14.1	HCA^b^	386/195/177	328/234
8	14.4	6,8-di-C-Glucosylapigenin^a^	595/577/559/541	271/335
9	16.1	HCA^b^	386/195/177	328/234
10	18.5	Unknown	595	
11	19.4	Unknown	573	
12	19.5	2′′-*O*-xylosylvitexin^a^	565/433/415/397	268/337
13	20.2	Unknown	565	
14	20.3	Unknown	595	
15	20.5	Flavanone-*O*-rutinoside^b^	611/465/303	
16	21.2	Luteolin-7-*O*-rutinoside^a^	595/595/449/287	255/266sh/348
17	22.1	8-*C*-glucosyldiosmetin^a^	463/445/427	254/266/348
18	24.4	Flavone-*O*-rutinoside^b^	609/463/301	
19	25.8	Diosmin^a^	609/463/301	253/267/347
20	26.3	Hesperidin^a, PCA^	611/465/449/303	283/331
21	29.1	Flavanone-*O*-rutinoside^b^	611/465/303	
22	32.2	Isosakuranetin rutinoside^a^	595/449/287	283/331
23	33.5	Unknown	728	
24	33.5	Unknown	359	
25	34.1	Unknown	713	
26	34.4	Unknown	359	
27	35.2	Unknown	359	
28	35.3	Unknown	331	
29	36.1	Isosinensetin^a^	373	249/270/342
30	37.1	DesmethylPMF^b^	389	
31	37.6	Sinensetin^a, PCA^	373	243/264/333
32	38.4	DesmethylPMF^b^	345	
33	39.4	Nobiletin^a^	403	249/270/334
34	40.1	Tetramethyl-*O*-Scutellarein^a^	343	265/322
35	40.2	Unknown	375	
36	40.5	Heptamethoxyflavone^a^	433	254/270sh/341
37	41.3	DesmethylPMF^b^	359	
38	41.5	Tangeretin^b^	373	271/321
39	42.3	DesmethylPMF^b^	359	
40	43.2	5-Desmethylnobiletin^a^	389	

^a^Identified by matching their retention time, mass spectra, and UV spectra with known standard. The UV spectra were further compared to earlier published spectra [18].

^b^Tentatively identified using +APCI-MS fragmentation patterns.

**Figure 3:**
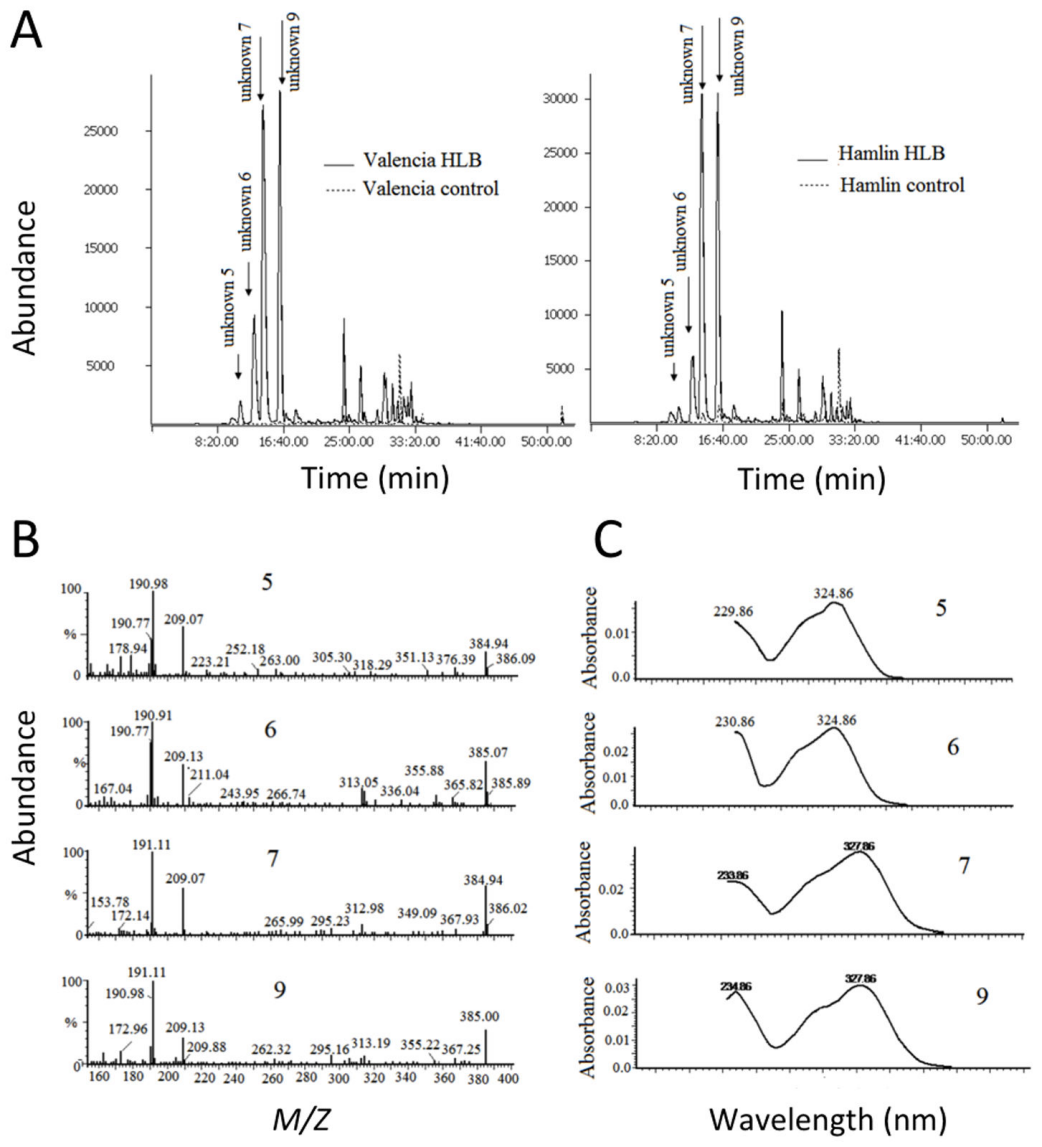
Typical APCI-HPLC-MS chromatograms of ‘Hamlin’ and ‘Valencia’ leaf extracts and mass-spectra and UV spectra of unknown 5, 6, 7, and 9. A) selective ion recording chromatograms of “*m/z*” specie of 177 in the positive ion mode; **B**) negative electrospray ionization mass spectra ; **C**) UV spectra of unknown **5**, **6**, **7**, and **9**.

Also present in the leaf metabolite profiles of the ‘Hamlin’ and ‘Valencia’ seedlings were compounds with spectroscopic properties indicative of a set of structurally-related HCAs. Compounds **5**–**7** and **9** (Figure 3A) each exhibited a mass spectra and a UV spectra (Figure 3B-C) and fluorescence emission spectra (data not shown) similar to feruloyl-derivatives of galactaric and glucaric (aldaric) acids reported in sweet orange peels [21,22], rye leaves [23], and cowpeas [24]. In cowpeas, six isomeric forms of trans-feruloylaldaric acid have been identified. Compounds **5**–**7** and **9** exhibited fragment ions at 177 *m/z* and 195 *m/z m/z* (Table 3) with positive electrospray ionization (ESI) and atmospheric pressure chemical ionization (APCI), while with negative ESI, fragment ions at 191, 209, and 385 *m/z* were additionally observed (Figure 3B). The 177 and 195 *m/z* ions are indicative of fragment ions generated from ferulic acid (FA) [FA-H_2_O+H] ^+^ and [FA+H]^+^ ions, respectively [21]. The 191 *m/z* ion fragments observed with negative ESI likely originated from [galactaric acid (GalA)-H_2_O-H]^-^ or [glucaric (GluA) acid-H_2_O-H]^-^ moieties, the 209 *m/z* from [GalA-H]^-^ or [GluA-H]^-^, and the 385 *m/z* ions from the ionized molecular ions [FA GalA-H]^-^ or [FA GluA-H]^-^ [24]. Ferulic acid-containing alkaloid feruloylputrescine, compound **4**, was also detected with positive ESI and APCI [25,26,27].

### Effect of *C*las infection on sweet orange leaf metabolites

**Figure 4:**
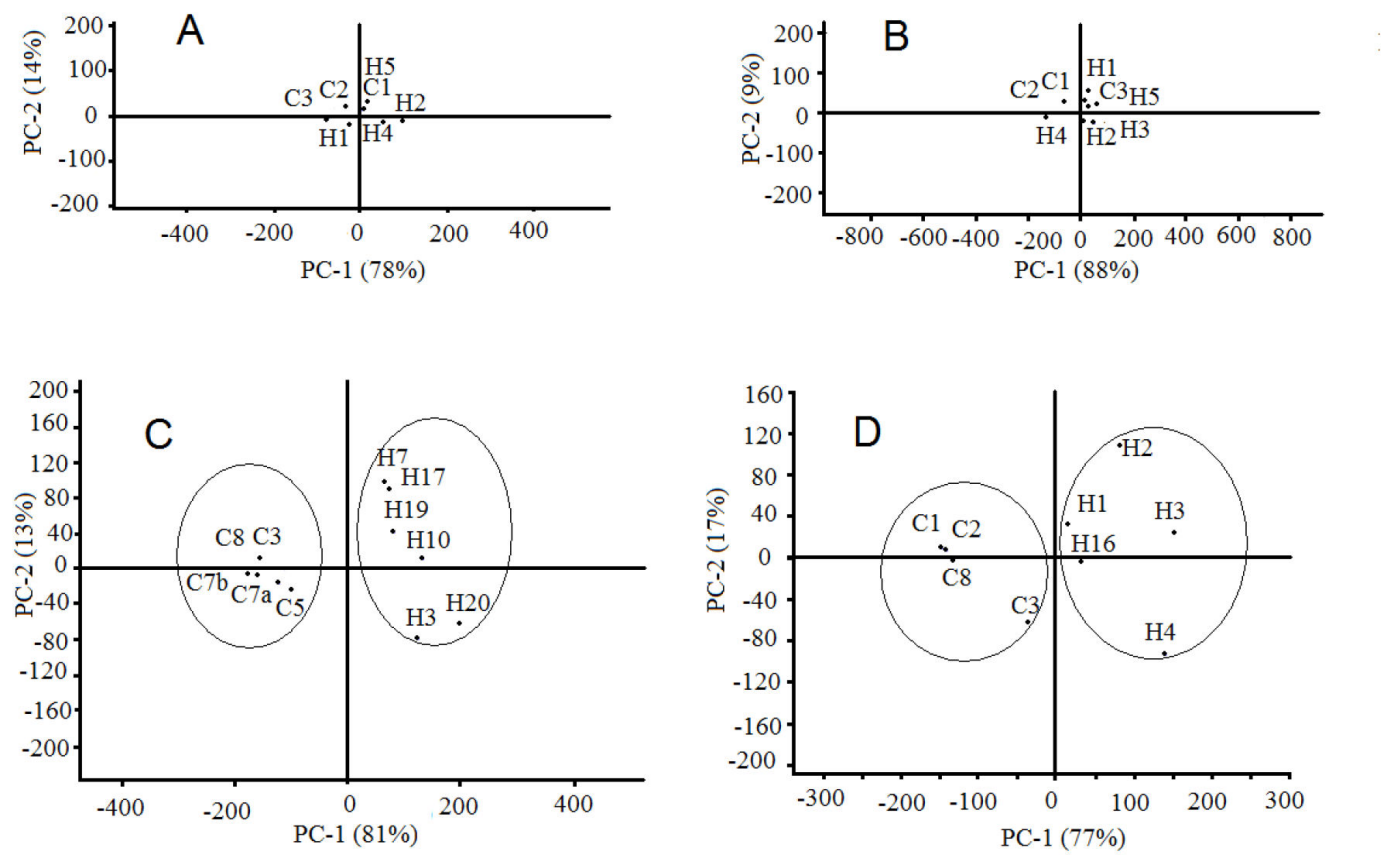
Principal component analysis of HPLC-MS leaf metabolites from ‘Valencia’ and ‘Hamlin’ trees. **A**) score plot of leaf metabolites from ‘Valencia’ trees 3 weeks after inoculation; **B**) score plot of leaf metabolites from ‘Hamlin’ trees 3 weeks after inoculation; **C**) score plot of leaf metabolites from ‘Valencia’ trees 38 weeks after inoculation; **D**) score plot of leaf metabolites from ‘Hamlin’ trees 38 weeks after inoculation.

Principal component analysis (PCA) and the *t*-test were carried out on each set of leaf samples to compare the metabolite profiles of the control to those from *C*las-inoculated trees and to identify compounds whose relative amounts were significantly affected by the progression of HLB disease. The PCA and *t*-test are the most common statistical analysis approach used in untargeted large-scale plant metabolomics studies [28,29]. No group clustering was observed in the score plots from PCA of ‘Valencia’ leaf metabolites during the first 23 weeks (data not shown). However, at 27 weeks, clustering into two groups (HLB and control) began to form (data not shown), and the clustering was well-defined by 38 weeks (Figure 4C). Loading values from PCA for the first or second principal components (Table 4) of compound **6**, **7**, **9**, and **20**, were greatest, suggesting that they were responsible for the group clustering. 

**Table 4:** Loadings from PCA for metabolites in leaves from ‘Valencia’ or ‘Hamlin’ at sampling dates when group clustering was observed^a^.

Compound		Loadings				
		Time after inoculation (week)				
		27	29	35	35	38
		‘Valencia’			‘Hamlin’	
PC-1	**7**	**0.24**			**0.15**	**0.35**
	9					0.27
	19				0.19	
	**20**	**0.82**	**0.91**	**0.89**	**0.93**	**0.81**
	31	0.28	0.21	0.21		
	33				-0.15	-0.25
	34	0.25				
	37	0.22				
	38				-0.10	
PC-2	**6**		**0.26**		**0.23**	**0.24**
	**7**		**0.54**	**0.67**	**0.74**	**0.67**
	**9**		**0.42**	**0.58**	**0.47**	**0.49**
	**20**	**0.54**	**0.21**	**0.29**	**-0.24**	**-0.47**
	33		0.48			
	38				-0.22	
	40		0.25			

^a^Compounds relevant to both varieties were bolded.

Similar score plots from PCA of ‘Hamlin’ leaves provided evidence of clustering into two groups (HLB and control) 35 weeks after grafting (data not shown) and the clustering was well-defined at 38 weeks (Figure 4D). Cluster regions were arbitrarily drawn in Figure 4C-D to help visualization. As in the changes that occurred in ‘Valencia’ leaves, compounds **6**, **7**, **9**, **18**–**20**, **31**, and **37** were the main compounds responsible for group clustering in ‘Hamlin’ leaves. 

Comparison of means of metabolite normalized area (relative amounts) from HLB-inoculated *versus* control ‘Valencia’ leaves revealed no significant increase in any metabolite at 3 and 23 weeks after inoculation (Table 5). However, comparison of means of metabolite relative amounts between weeks 27 and 35 revealed that at one or more sampling dates 13 metabolites were five times higher in the HLB-affected leaves than in the control leaves, and four compounds were five-fold higher in the HLB-affected leaves at two sampling dates. These compounds are highlighted on Table 5. In particular, the relative amount of compound **13** was 58.7 and 46.3 times higher in the HLB-affected leaves in weeks 29 and 35, respectively; the relative amount of compound **6** was 13.0 and 25.8 times higher in weeks 27 and 29, respectively, and compound **7** was 17.2 and 11.1 times higher in weeks 27 and 29, respectively.

**Table 5:** The relative amounts of metabolites from leaves of *C*Las-inoculated ‘Valencia’ trees with respect to control ‘Valencia’ trees.

Peak no.	Compound	Week 3	Week 23	Week 27	Week 29	Week 35	Week 38	Time effect (*p-*value)^a^	Group effect (*p-*value)^a^
1	Unknown	0.7	0.9	**9.8**	1.5	1.4	**22.6**	0.3940	0.0380
2	Unknown	0.9	1.5	1.5	1.2	1.0	**1.7**	0.0060	0.2010
3	Unknown	1.0	0.7	3.6	1.3	1.2	**2.3**	0.3780	0.0290
4	Feruloylputrescine	1.6	1.0	2.0	2.0	3.3	**12.2**	0.0820	0.0056
5	HCA	1.6	1.3	**10.5**	**6.4**	3.2	**127.7**	0.1280	0.0003
6	HCA^PCA^	1.5	3.3	**13.0**	**25.8**	291.2	**158.3**	0.0056	<0.0001
7	HCA^PCA^	2.3	2.2	**17.2**	**11.1**	105.6	**48.8**	0.0007	<0.0001
8	6,8-di-C-Glucosylapigenin	1.3	0.7	4.7	**2.4**	**9.9**	**4.5**	0.2000	<0.0001
9	HCA^PCA^	1.3	2.0	6.8	**9.3**	45.8	**24.1**	0.0064	<0.0001
10	Unknown	1.3	2.2	NA	6.5	114.8	**31.0**	0.0206	0.0078
11	Unknown	0.9	2.7	12.3	31.8	4.0	**273.6**	0.0087	0.0003
12	2′′-*O*-xylosylvitexin	0.9	1.0	7.2	202.4	81.9	**86.6**	0.0381	0.0147
13	Unknown	1.2	12.5	3.2	**58.7**	**46.3**	**26.2**	0.0104	0.0008
14	Unknown	1.4	1.0	NA	NA	**10.2**	**4.5**	0.0513	0.0009
15	Flavanone-*O*-rutinoside	0.6	1.4	15.4	12.7	10.3	**0.3**	0.0200	0.7039
16	Luteolin-7-*O*-rutinoside	1.4	2.8	10.9	2.2	1.5	1.3	0.0413	0.0383
17	8-*C*-glucosyldiosmetin	1.4	14.8	11.0	3.6	**6.5**	**20.0**	0.6120	0.0002
18	Flavone-*O*-rutinoside	1.1	1.7	2.3	1.6	**5.3**	**2.0**	0.2540	0.0058
19	Diosmin	1.2	1.3	4.3	1.6	2.2	**2.4**	0.9260	0.0020
20	Hesperidin^PCA^	1.2	1.6	**4.1**	2.2	1.8	**7.5**	0.1860	0.0006
21	Flavanone-*O*-rutinoside	1.4	3.6	5.0	0.4	2.3	**10.7**	0.4840	0.1179
22	Isosakuranetin rutinoside	1.2	1.8	**5.5**	2.9	3.4	**8.2**	0.2003	< 0.0001
23	Unknown	1.0	1.0	3.9	2.9	1.8	**16.6**	0.1849	0.0024
24	Unknown	0.8	1.0	5.4	**4.9**	2.0	**6.4**	0.8840	0.0003
25	Unknown	0.9	0.8	3.4	**5.7**	2.8	**227.2**	0.0001	0.0095
26	Unknown	**0.8**	1.2	4.6	**4.0**	2.6	**1.9**	0.0520	0.0041
27	Unknown	0.7	0.6	3.2	**4.2**	3.5	1.3	0.1740	0.1055
28	Unknown	0.8	1.2	4.9	**7.7**	2.1	**3.1**	0.3660	0.0002
29	Isosinensetin	**0.7**	0.4	0.8	1.1	1.5	**0.2**	0.4450	0.0029^b^
30	DesmethylPMF	**0.7**	0.7	1.8	**3.4**	**4.2**	0.8	0.6540	0.5315
31	Sinensetin^PCA^	**0.8**	1.2	4.8	**4.7**	2.4	**10.3**	0.0001	0.0037
32	DesmethylPMF	0.7	1.4	4.8	**2.9**	2.9	**19.3**	0.0019	0.0030
33	Nobiletin	**0.8**	0.6	1.5	2.2	1.9	**0.6**	0.2510	0.4680
34	Tetramethyl-*O*-Scutellarein^PCA^	0.9	1.1	6.0	**4.3**	2.0	**2.0**	0.0223	0.0082
35	Unknown	0.7	0.6	2.1	2.6	3.4	0.7	0.4580	0.7399
36	Heptamethoxyflavone	0.7	0.9	3.1	2.6	2.7	0.8	0.1890	0.2531
37	DesmethylPMF^PCA^	0.9	1.5	5.7	**2.9**	2.4	**14.3**	0.0136	0.0003
38	Tangeretin^PCA^	0.9	0.7	2.1	**2.8**	2.2	1.0	0.2340	0.1070
39	DesmethylPMF	0.8	0.5	1.0	1.2	1.4	**0.3**	0.2018	0.0117^b^
40	5-Desmethylnobiletin	0.8	0.6	1.4	2.6	2.3	**0.7**	0.2590	0.8380

Bolded ratio means that the averages of these compounds in *C*Las-inoculated trees (n = 5) were significantly different (*p*-value < 0.05) from the controls (n = 3).

^a^ *p*-value for group effect and time effect for the whole model (all sampling dates) using ANCOVA analysis.

^b^The average of the relative amount of these compounds in all sampling dates was significantly lower in *C*Las-inoculated plants. *P*-values (in the same column) lower than 0.05 without any superscript are higher in *C*Las-inoculated plants.

^PCA^ Compound with loading > 0.2 from principal component analysis, hence, accounts for group clustering.

NA means that the level of the compound was below the detection limit.

**Figure 5:**
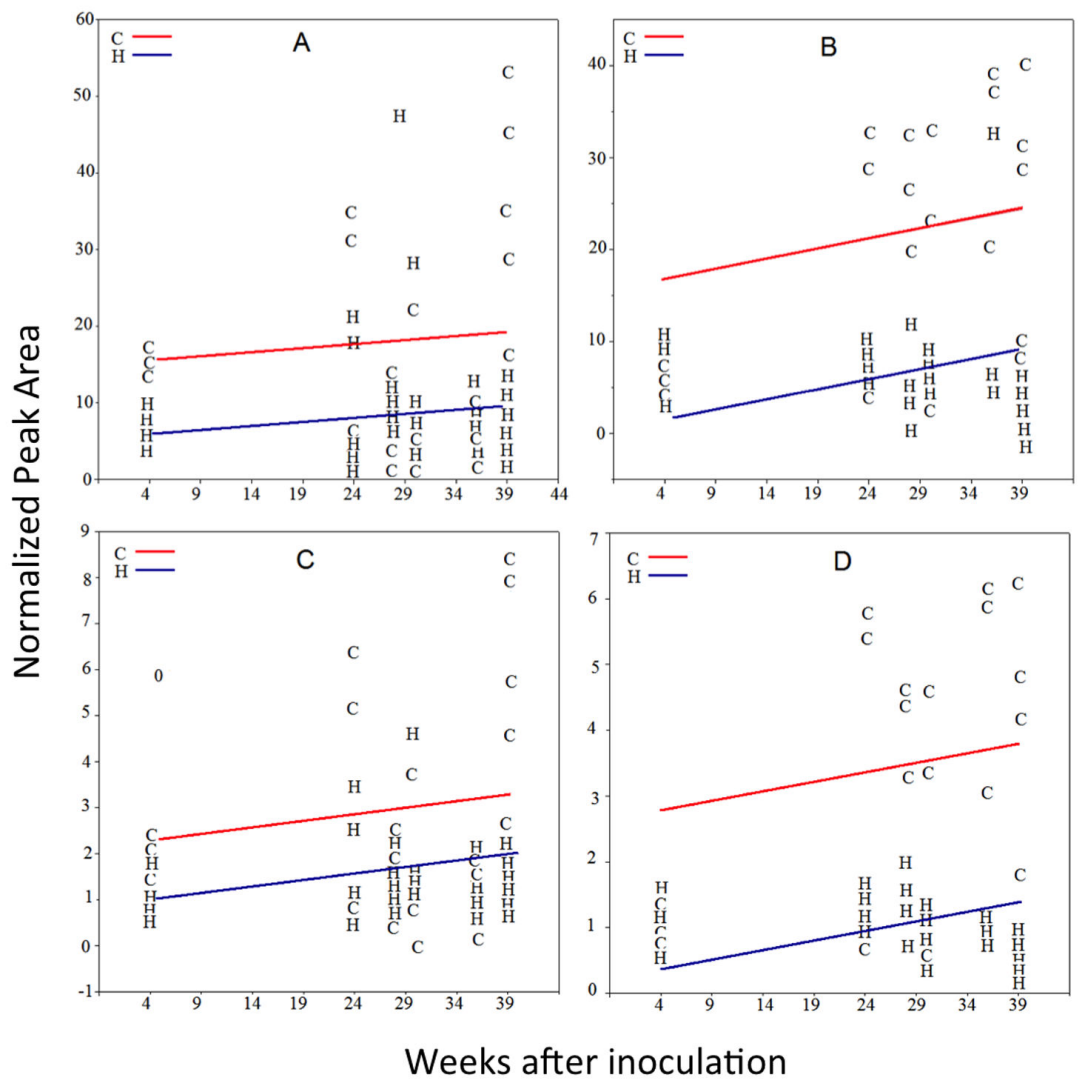
Relative amounts versus time (week) of different citrus leaf metabolites from controls and *C*Las-inoculated trees. **A**) unknown **29** in ‘Valencia’ leaves; **B**) unknown **29** in ‘Hamlin’ leaves; **C**) unknown **39** in ‘Valencia’ leaves; **D**) unknown **39** in ‘Hamlin’ leaves.

In ‘Hamlin’ trees, compounds **5**, **16**, **22**, **27**, and **32** occurred at significantly higher levels at week 3 in leaves of *C*las-inoculated trees than in control leaves (Table 6). Because the ratios of these compounds in *C*las-inoculated trees were slightly higher than those from the control and none of these compounds was significantly higher at 23 weeks after inoculation, these differences are not likely to be caused by *C*las infection. Slight variation in leaf metabolite levels could result from differences in leaf maturity and leaf age. At week 23, there was no significant difference in any leaf metabolite, and only a slight increase (20%) in the relative amount of compound **2** in leaves from *C*las-inoculated ‘Hamlin’ trees was observed 2 weeks later. At week 38, where the leaf symptoms were clearly evident, dramatic increases in the numbers of compounds with significantly different means between the HLB-affected and control leaves occurred. The number of compounds that was induced in the HLB-affected ‘Hamlin’ leaves was less than those induced in the HLB-affected ‘Valencia’ leaves, yet those compounds observed in the HLB-affected ‘Hamlin’ leaves matched the compounds found at earlier dates in ‘Valencia’ trees. In agreement with the later development of visual leaf symptom in the *C*las-infected trees, changes in metabolite profiles appeared later in ‘Hamlin’ leaves than those in ‘Valencia’. Table 6 highlights the metabolites that were higher in leaves from *C*las-infected ‘Hamlin’ plants than in leaves from controls. The flavone glycoside (17) was 32 times more concentrated at week 35, and the putative HCAs (6 and 7) were more than 10 times in leaves from HLB-infected ‘Hamlin’ trees 38 weeks after inoculation. A number of the PMFs and PMF-derivatives, **29**, **36**, and **39** occurred at significantly lower levels in leaves of the HLB-affected trees at week 27, and compounds **29**, **33**, **36** , and **39** occurred at significantly lower levels in leaves of HLB-affected trees at week 38 (Table 6). The abundance of compounds **29** and **39** against time in control and *C*las-inoculated trees is shown in Figure 5. Decreased levels of PMFs were similarly reported in field-grown HLB-symptomatic ‘Valencia’ and ‘Midsweet’ orange leaves [14].

**Table 6:** The relative amounts of metabolites from *C*Las-inoculated ‘Hamlin’ trees with respect to control ‘Hamlin’ trees.

Peak no.	Compound	Week 3	Week 23	Week 27	Week 29	Week 35	Week 38	Time effect (*p-*value)^a^	Group effect (*p*-value)^a^
1	Unknown	1.2	0.6	9.2	1.5	41.5	1.0	0.9890	0.0900
2	Unknown	1.0	0.8	**1.2**	**2.1**	1.0	1.6	0.0050	0.7300
3	Unknown	1.3	0.7	1.3	1.4	2.1	1.2	0.0149	0.1673
4	Feruloylputrescine^a^	2.0	1.1	16.4	**7.9**	41.1	1.7	0.0210	0.0137
5	HCA	**8.7**	1.1	0.9	NA	1.0	**5.2**	0.0187	0.0059
6	HCA^PCA^	4.5	11.4	14.0	7.0	17.0	**33.0**	0.0128	0.0006
7	HCA^PCA^	4.1	43.3	4.3	4.8	88.9	**12.0**	0.0007	0.0001
8	6,8-di-C-Glucosylapigenin^a^	2.9	1.9	2.0	1.5	13.5	1.8	0.4298	0.0007
9	HCA^PCA^	2.6	14.5	2.8	3.2	39.3	**7.2**	0.0183	0.0001
10	Unknown	1.8	1.3	0.3	3.6	52.0	6.0	0.0907	0.0132
11	Unknown	2.0	8.1	36.2	4.2	146.8	**6.9**	0.0180	0.0005
12	2′′-*O*-xylosylvitexin	2.5	2.0	NA	4.5	355.3	23.6	0.0660	0.0194
13	Unknown	1.9	2.4	1.1	4.3	62.1	9.6	0.0936	0.0123
14	Unknown	1.6	1.4	NA	2.0	17.3	5.4	0.4280	0.0084
15	Flavanone-*O*-rutinoside	3.7	15.6	0.3	0.2	1.3	1.0	0.0180	0.1520
16	Luteolin-7-*O*-rutinoside	**3.2**	1.6	0.4	1.4	**3.6**	1.9	0.0952	0.0327
17	8-*C*-glucosyldiosmetin	2.5	1.5	4.4	2.4	**32.0**	2.6	0.8600	0.0017
18	Flavone-*O*-rutinoside	1.5	3.0	1.3	1.3	2.4	2.9	0.0605	0.0047
19	Diosmin	1.7	0.9	1.2	1.5	**3.7**	1.9	0.5138	0.0035
20	Hesperidin^PCA^	1.8	1.1	5.8	2.0	14.1	2.0	0.5210	0.0008
21	Flavanone-*O*-rutinoside	1.8	0.9	NA	2.2	11.9	2.3	0.7970	0.0018
22	Isosakuranetin rutinoside	**1.7**	0.1	NA	1.4	13.9	2.0	0.4800	0.7350
23	Unknown	1.1	1.6	4.7	1.3	7.8	2.1	0.0030	0.0010
24	Unknown	1.4	1.5	2.6	1.9	2.2	1.1	0.0932	0.0001
25	Unknown	0.9	2.4	130.0	2.3	225.1	2.4	0.0010	0.0039
26	Unknown	1.2	0.7	1.8	1.6	2.9	0.7	0.0724	0.2864
27	Unknown	**2.1**	0.8	0.7	1.7	1.4	0.5	0.6949	0.9887
28	Unknown	1.6	0.8	1.3	2.9	3.2	1.8	0.2536	0.0006
29	Isosinensetin	1.2	0.5	**0.2**	0.3	0.3	**0.2**	0.0951	0.0010^b^
30	DesmethylPMF	1.5	0.8	1.1	0.5	0.6	0.8	0.1669	0.4856
31	Sinensetin^PCA^	1.2	2.1	3.3	1.7	4.2	1.1	0.0010	0.0027
32	DesmethylPMF	**1.5**	1.3	2.6	1.9	15.6	1.7	0.0003	0.0003
33	Nobiletin	1.2	0.7	0.8	0.4	**0.4**	**0.4**	0.9670	0.0030^b^
34	Tetramethyl-*O*-Scutellarein^PCA^	1.1	1.2	1.6	2.1	11.6	1.4	0.0003	0.0023
35	Unknown	1.3	0.7	2.0	0.6	0.7	0.7	0.6110	0.9910
36	Heptamethoxyflavone	1.4	0.7	**0.4**	0.6	0.2	**0.2**	0.9960	0.0014^b^
37	DesmethylPMF^PCA^	1.1	1.8	3.7	2.1	6.0	1.5	0.0043	0.0010
38	Tangeretin^PCA^	1.1	0.7	1.9	0.5	1.5	**0.5**	0.5310	0.0013^b^
39	DesmethylPMF	1.1	0.5	**0.3**	0.5	0.4	**0.2**	0.1100	0.0001^b^
40	5-Desmethylnobletin	1.1	0.6	1.0	0.4	**0.3**	0.4	0.1530	0.0260

Bolded ratio means that the averages of these compounds in *C*Las-inoculated trees (n = 5) were significantly different (*p*-value < 0.05) from the controls (n = 3).

^a^ *p*-value for group effect and time effect for the whole model (all sampling dates) using ANCOVA analysis.

^b^The average of the relative amount of these compounds in all sampling dates was significantly lower in *C*Las-inoculated plants. *P*-values (in the same column) lower than 0.05 without any superscript are higher in *C*Las-inoculated plants.

^PCA^ Compound with loading > 0.2 from principal component analysis, hence, accounts for group clustering.

NA means that the level of the compound was below the detection limit.

**Figure 6:**
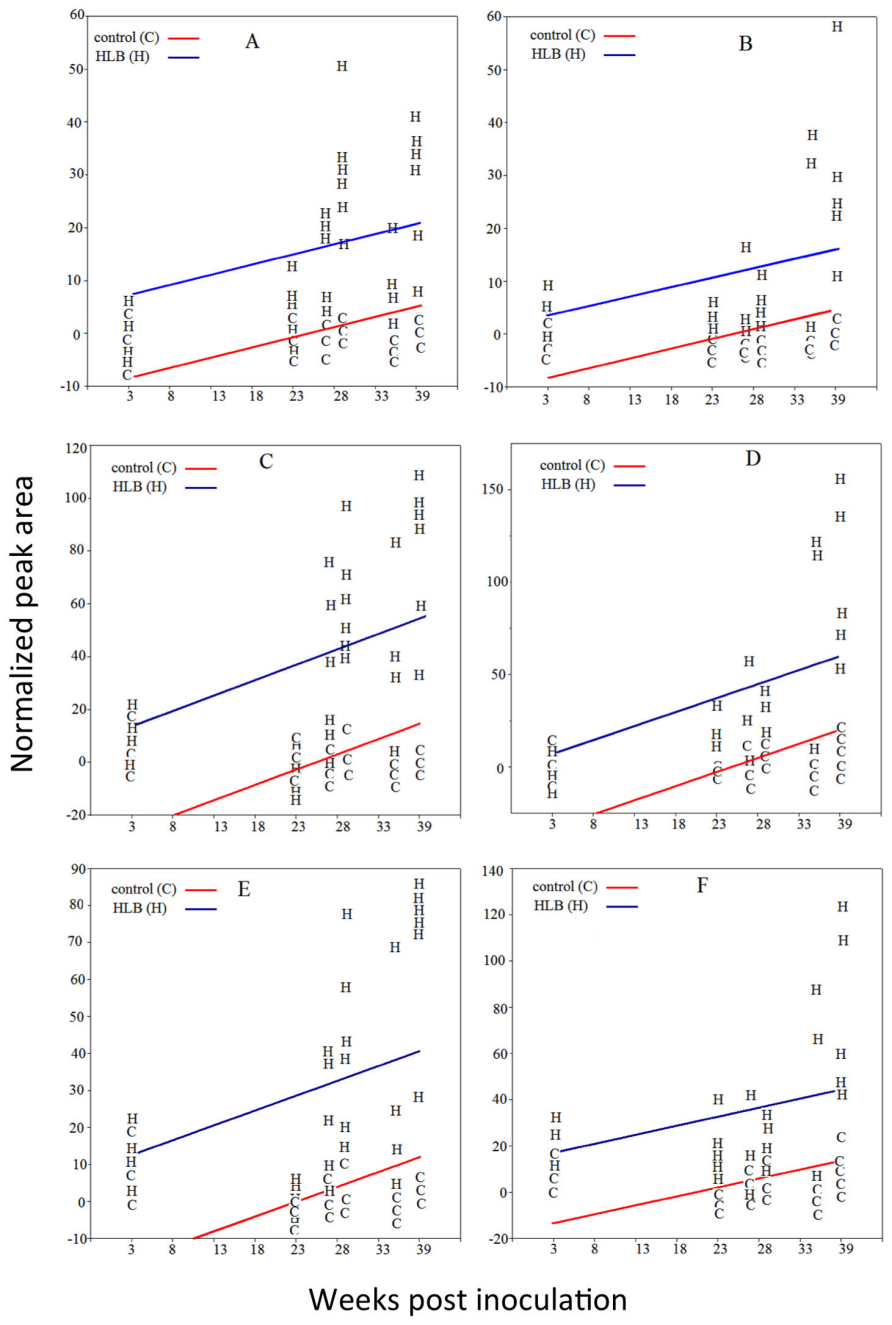
Relative amounts versus time (week) of different citrus leaf metabolites from controls and *C*Las-inoculated trees. **A**) unknown **6** in ‘Valencia’ leaves; **B**) unknown **6** in ‘Hamlin’ leaves; **C**) unknown **7** in ‘Valencia’ leaves; **D**) unknown **7** in ‘Hamlin’ leaves; **E**) unknown **9** in ‘Valencia’ leaves; F) unknown **9** in ‘Hamlin’ leaves.

The *t*-test results support the findings made by PCA, particularly pertaining to the putative HCAs, **6**, **7**, and **9**. As shown in Figure 3A and Figure 6, the levels of these compounds were higher in leaves from *C*las-infected ‘Valencia’ and ‘Hamlin’ trees, and their levels increased with time and symptoms progression. These elevated levels of HCAs in the HLB-affected trees are consistent with findings reported earlier with mature field-grown ‘Valencia’ and ‘Midsweet’ orange trees [14]. At week 3, the levels of these putative HCAs in leaves from HLB-infected trees were equal to those from controls. However, the levels of these compounds were higher in leaves from *C*las-infected trees at 27, 29, 35, and 38 weeks after inoculation.

ANCOVA analysis (columns 8 and 9 in Table 5 and Table 6) showed that most of the detected leaf metabolites were significantly affected by *C*las infection. The use of ANCOVA analysis was necessary to account for the effect of the covariant (time) and to reduce the error that was caused by the none-even distribution of plant response. ANCOVA analysis showed that time effect was significant in the model, especially for those metabolites that were significantly affected by *C*las infection. The ANCOVA analysis showed that 28 compounds were significantly higher in leaves from *C*las-inoculated ‘Valencia’ plants, 2 metabolites (29 and 39) were lower, and 10 were not affected. Similar effects were also observed in ‘Hamlin’ leaf metabolites. Twenty-seven metabolites were higher in leaves from *C*las-inoculated ‘Hamlin’ trees, five metabolites were lower (29, 33, 36, 38, and 39), and eight metabolites were not affected.

The results from the ANCOVA analysis were consistent with *t*-test and PCA results. For example, the *t*-test showed that ratios of the relative abundance of metabolite **29** in leaves from *C*las-inoculated plants were always less than 1 at one or more sampling dates (Table 5 and Table 6). The ANCOVA analysis of the whole data set for ‘Hamlin’ and ‘Valencia’ leaves also showed that compound **29** was significantly lower in *C*las-inoculated plants (Table 5 and Table 6 and Figure 5). Metabolites **6**, **7**, and **9** are another example. The *t*-test showed that the mean of these metabolites in *C*las-inoculated trees was significantly higher than those in the controls at one or more sampling dates (Table 5 and Table 6). In agreement with the *t*-test results, the ANCOVA analysis showed that these compounds were also higher in *C*las-infected plants (Table 5 and Table 6 and Figure 6). In addition, the ANCOVA analysis confirmed the PCA results. For example, the PCA analysis showed that unknowns **6**, **7**, and **9** were responsible for group clustering and the ANCOVA analysis showed that these metabolites were significantly higher in *C*las-inoculated trees (*p*-value < 0.001).

In agreement with our results, change in secondary metabolites has been observed in many plants after pathogen infection. A significant increase in concentrations of phenolic compounds was observed in xylem tissues of grapevines 2 months after inoculation with *Xylella fastidiosa* [30]. The increase in phenolic compounds suggested that the cell wall of the infected plant becomes thicker and more resistant to degradation by *Xylella fastidiosa* [30]. Higher levels of several amino acids, phenolic compounds, reducing sugars, and defense enzymes were also observed in ‘*Candidatus* Liberibacter solanacearum’-infected potato tubers [4]. Tubers that were infected 5 weeks before harvest showed higher levels than those infected one week before harvest. No correlation between elevated compounds and ‘*Candidatus* Liberibacter solanacearum’ titer was observed except for phenolics. However, there was a strong association between biochemical response and symptom severity [4].

Our results showed that hydroxycinnamate compounds were induced in leaves from ‘Hamlin’ and ‘Valencia’ sweet orange trees in response to *C*las infection. In fact, accumulation of phenylpropanoids (hydroxycinnamic acid derivatives) was also observed in tomato plant infected with the bacterial pathogen *Pseudomonas syringae* pv. *Tomato* [31,32]. In addition, an accumulation of *p*-coumaric, caffeic, and ferulic acids mainly forming esters with glucose has been observed in cucumber infected with prunus necrotic ringspot virus or melon infected with melon necrotic spot virus [33]. In agreement with the induction of the phenylpropanoids observed, both viral infections studied induced the expression of cinnamic acid 4-hydroxylase (C4H) and phyenylalanine ammonia-lyase (PAL) genes. Because an increase of *p*-coumaric, caffeic, and ferulic acids was also observed in leaves from cucumber treated with powdery mildew [34], Bellés et al. [33] suggested that accumulation of HCAs is involved in plant defense against different biotic stresses.

The involvement of feruloyl HCAs as components of plant responses against microbial attacks has been previously studied [31,35]. The molecular actions of HCAs in plant defenses are not well-defined, but it has been proposed that they can be incorporated into the plant cell wall to strengthen it against microbial attack, can act as direct antimicrobial agents, or may act as signal molecules [36]. HCAs are produced by the activation of the phenylpropanoid biosynthetic pathway which is induced in response to biotic and abiotic stress [31]. The first step in the phenylpropanoid pathway is the conversion of phenylalanine to trans-cinnamic acid (phenylpropenoic acid) by phenylalanine ammonia-lyase (PAL) [31]. In the second step, cinnamic acid is being converted to 4-hydroxycinnamic acid by cinnamate 4-hydroxylase (C4H) [31]. The hydroxycinnamic acids (*p*-coumaric and ferulic) serve as a precursor for many phenylpropanoid derivatives with antimicrobial function [31].

In conclusion, this study showed that sweet orange leaf metabolites were significantly affected by *C*las infection. The earliest shift in metabolite profiles of leaves from *C*las-infected trees was noticed 27 weeks after inoculation. Most of the detected metabolites increased with time, while few decreased. The abundance of some metabolites like HCAs increased more than 10-fold in leaves from *C*las-infected trees, whereas the abundance of some metabolites like isosinensetin and desmethylPMF decreased more than 3-fold. The increase in HCAs and other metabolite levels in the leaves of *C*las-infected plants may be potentially linked to sweet orange’s response to the *C*las infection. Further studies are needed to examine the effects of *C*las infection on both resistant and susceptible cultivars to test whether the induced metabolites are related with plant resistance.
